# Chinese medicine PaBing-II protects human iPSC-derived dopaminergic neurons from oxidative stress

**DOI:** 10.3389/fimmu.2024.1410784

**Published:** 2024-08-02

**Authors:** Shouhai Wu, Cuiping Rong, Ruishan Lin, Kaiyuan Ji, Tongxiang Lin, Weimin Chen, Wei Mao, Yang Xu

**Affiliations:** ^1^ State Key Laboratory of Dampness Syndrome of Chinese Medicine, The Second Affiliated Hospital of Guangzhou University of Chinese Medicine, Guangdong, Guangzhou, China; ^2^ Department of Nephrology, Guangdong Provincial Hospital of Chinese Medicine, Guangzhou, China; ^3^ Laboratory of Molecular Biology, The First Affiliated Hospital of Guangxi University of Chinese Medicine, Guangxi, Nanning, China; ^4^ Experimental Teaching Center, School of Basic Medical Sciences, Guangzhou University of Chinese Medicine, Guangdong, Guangzhou, China; ^5^ Guangzhou Women and Children’s Medical Center, Guangzhou Medical University, Guangdong, Guangzhou, China; ^6^ College of Animal Sciences, Fujian Agriculture and Forestry University, Fujian, Fuzhou, China; ^7^ Department of Cardiology, Heart Regeneration and Repair Key Laboratory of Zhejiang Province, State Key Laboratory of Transvascular Implantation Devices, The Second Affiliated Hospital, Zhejiang University School of Medicine, Hangzhou, China; ^8^ Research Center for Life Science and Human Health, Binjiang Institute of Zhejiang University, Zhejiang, Hangzhou, China

**Keywords:** Parkinson’s disease, hiPSCs, DAn, Nrf2/ARE, ROS, inflammation

## Abstract

**Background:**

PaBing-II Formula (PB-II) is a traditional Chinese medicine for treating Parkinson’s disease (PD). However, owing to the complexity of PB-II and the difficulty in obtaining human dopaminergic neurons (DAn), the mechanism of action of PB-II in PD treatment remains unclear. The aim of this study was to investigate the mechanisms underlying the therapeutic benefits of PB-II in patients with PD.

**Methods:**

hiPSCs derived DAn were treated with H_2_O_2_ to construct the DAn oxidative damage model. SwissTargetPrediction was employed to predict the potential targets of the main compounds in serum after PB-II treatment. Metascape was used to analyze the pathways. Sprague-Dawley rats were used to construct the 6-hydroxydopamine (6-OHDA)-induced PD model, and the duration of administration was four weeks. RNA sequencing was used for Transcriptome analysis to find the signal pathways related to neuronal damage. The associated inflammatory factors were detected by enzyme-linked immunosorbent assay (ELISA). We identified PB-II as an Nrf2 activator using antioxidant-responsive element luciferase assay in MDA-MB-231 cells.

**Results:**

*In vitro* experiments showed that the treatment of PB-II-treated serum increased the percentage of TH^+^ cells, decreased inflammation and the apoptosis, reduced cellular reactive oxygen species, and upregulated the expression of Nrf2 and its downstream genes. Pathway analysis of the RNA-seq data of samples before and after the treatment with PB-II-treated serum identified neuron-associated pathways. *In vivo* experiments demonstrated that PB-II treatment of PD rat model could activate the Nrf2 signaling pathway, protect the midbrain DAn, and improve the symptoms in PD rats.

**Conclusion:**

PB-II significantly protects DAn from inflammation and oxidative stress via Nrf2 pathway activation. These findings elucidate the roles of PB-II in PD treatment and demonstrate the application of hiPSC-derived DAn in research of Chinese medicine.

## Graphical Abstract

PaBing-II Formula (PB-II) is a traditional Chinese medicine for treating Parkinson’s disease (PD). To investigate the mechanisms underlying the therapeutic benefits of PB-II in patients with PD, we developed human dopaminergic neurons (DAn) oxidative damage models by treating human induced pluripotent stem cells (hiPSCs) derived DAn with H_2_O_2_ as well as the 6-hydroxydopamine (6-OHDA)-induced PD rat model. Through *in vitro* and *in vivo* studies, we demonstrate that PB-II significantly protects DAn from oxidative stress and inflammation via Nrf2 pathway activation, and is manifested as a treatment for Parkinson’s disease.

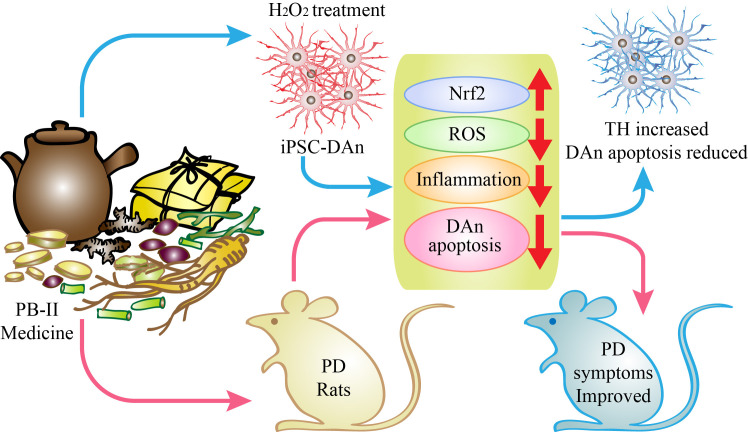

## Introduction

Parkinson’s disease (PD) is a common progressive neurodegenerative disease characterized by tremors and bradykinesia (1). As patients age, their symptoms worsen (2). The hallmarks of PD are the degeneration of dopaminergic neurons (DAn) in the substantia nigra (SN) and the presence of Lewy bodies (2, 3). Although the mechanisms underlying PD pathology remain unclear, extensive evidence from postmortem brain tissue suggests that oxidative stress and deficiency of complex I activity and inflammation are related to PD pathogenesis (4–6). There is currently no cure for PD. The primary treatment for PD is dopamine supplementation or promotion of endogenous dopamine release. Levodopa, commonly used in PD treatment, can significantly improve the motor symptoms of PD; however, it cannot prevent disease progression and eventually leading to the death of DAn. Long-term use of Levodopa can also cause serious complications such as movement disorders, insomnia, and anxiety (7).

The PaBing-II Formula (PB-II), a Traditional Chinese Medicine, was developed and has been used to treat PD at the Second Affiliated Hospital of Guangzhou University of Chinese Medicine for over 20 years. The composition of PB-II is listed in **Table 1**. PB-II has significant therapeutic effects on patients with PD, particularly during the early stages (8, 9). PB-II can relieve the motor and non-motor symptoms of patients with PD, improve the therapeutic effects of dopaminergic drugs, reduce drug side effects, and improve their quality of life (10, 11). Previous studies have reported that gavaging rats with PD induced by 6-hydroxydopamine (6-OHDA) with PB-II improved rotational behavior (12), and protected DAn from apoptosis (13, 14). 6-OHDA can damage DAn in the SN of the midbrain, leading to PD symptoms in mice. The unilaterally 6-OHDA-lesioned rat model of PD has been invaluable in advancing our understanding of the mechanisms underlying parkinsonian symptoms and is widely used in PD research (15). However, the effects of PB-II on human DAn and the mechanisms underlying its therapeutic benefits remain unclear.

**Table 1:** Composition of PB-II.

Latin name	Family	The part used	Proportion
*Prunus mume* (Siebold) Siebold & Zucc	*Rosaceae*	Fruit	20 g
*Coptis chinensis* Franch.	*Ranunculaceae*	Rhizome	3 g
*Paeonia lactiflora* Pall.	*Paeoniaceae*	Root	20 g
*Angelica sinensis* (Oliv.) Diels	*Apiaceae*	Root	10 g
*Aconitum carmichaelii* Debeaux	*Ranunculaceae*	Root	10 g
*Rehmannia glutinosa* (Gaertn.) DC.	*Plantaginaceae*	Root	10 g
*Reynoutria multiflora* (Thunb.) Moldenke	*Polygonaceae*	Root	20 g
*Ligusticum striatum* DC.	*Apiaceae*	Rhizome	10 g
*Pueraria lobata* (Willd.) Ohwi	*Leguminosae*	Root	20 g
*Panax ginseng* C.A.Mey.	*Araliaceae*	Root and Rhizome	10 g
*Acorus tatarinowii* Schott	*Acoraceae*	Rhizome	5 g
*Gastrodia elata* Blume	*Orchidaceae*	Rhizome	10 g
*Chinemys reevesii* (Gray)		Carapax and plastron	10 g
*Glycyrrhiza uralensis* Fisch.	*Leguminosae*	Root and Rhizome	3 g

The names correspond to the latest version in The Plant List (http://www.theplantlist.org) or the Chinese Pharmacopoeia 2020 edition.

Because of the ability of induced pluripotent stem cells (iPSCs) to differentiate into all cell types in the body, iPSC technology is an essential tool for studying human diseases in dishes (16). In the present study, DAn were derived from hiPSCs and treated with H_2_O_2_ to establish DAn oxidation model. Using this model, the mechanisms underlying the therapeutic benefits of PB-II in PD patients were investigated.

## Materials and methods

### PB-II treated serum preparation

Refer to a previous report (12) regarding the original PB-II recipe. We prepared slices of Chinese crude drugs from the pharmacy of Guangdong Province Hospital of Chinese medicine, which were decocted twice with 10 and 8 times of water, filtered, and concentrated in a water bath at 80°C to 1.6 kg/L (measured by crude drug weight/volume). The rats were administered PB-II twice daily at 32 g/kg (crude drug weight/body weight). After gavaging for 2 weeks, the rats under 30 mg/kg pentobarbital sodium anesthesia were bled from the arterial blood and separated into medicated serum according to a previously reported method (17). The treated and control sera were inactivated at 56°C for 30 min and stored at -80°C before use.

### Liquid chromatography and mass spectrometry

LC-MS was conducted using a Dionex Ultimate 3000 UHPLC system (Thermo Fisher Scientific, Waltham, MA, USA) and a Q Exactive Orbitrap mass spectrometer. After screening, the analysis was performed using Waters (Milford, MA, USA) UPLCTM HSS T3 C18 (2.1 × 100 mm, 1.7 μm). Chromatographic conditions: Gradient elution was performed using acetonitrile (A) and -0.1% formic acid in water (B). The elution procedure was: 0 min, 10% A; 5 min, 20% A; 20 min, 60% A; 25 min, 90% A; 28 min 90% A; 29–33 min 10% A. The flow rate was 0.2 mL/min. Mass spectrometry conditions: Samples were ionized using an ESI ion source and analyzed using a Q Exactive Orbitrap high-resolution mass spectrometer. The main parameters of the ESI ion source were as follows: spray voltage 3500 V (Anion voltage, -3500 V); capillary temperature, 350°C; sheath gas, 40; auxiliary gas, 15. All other parameters were set to default values. A liquid eluent with a retention time of 0.5–29 min was selected for mass spectrometry analysis using an automatic switching valve.

### Network pharmacology analysis of PB-II treated serum

The targets were predicted using network pharmacology after identifying the main compounds in PB-II-treated serum. Probable targets of the identified compounds were predicted using SwissTargetPrediction (http://www.swisstargetprediction.ch/) (18). Targets with a probability score >0 were used to analyze the Gene Ontology (GO) functional annotations and Kyoto Encyclopedia of Genes and Genomes (KEGG) pathways using Metascape (https://metascape.org/gp/index.html#/main/step1) (19).

### Derivation of human DAn from iPSCs

In our previous studies, we established multiple iPS cell lines from human fibroblasts (20). This study selected an iPS cell line preserved in our laboratory for research. Based on our previous reports (21), DAn were differentiated from iPSC using the necessary media and related cytokines. iPSCs were plated at a density of 4 × 10^4^ cells/cm^2^ on Matrigel (BD)-coated tissue culture dishes for differentiation. Differentiation was performed in KSR medium. On day 3, the cells were nearly confluent (>80% confluency). The culture medium was gradually changed to N2 medium, supplemented on days 0–5 with SB431542 (5 μM) + LDN-193189 (100 nM) + Iwp2 (1 μM) to obtain retinal progenitors, and then split in a 1:3 ratio for the next six passages using Accutase. The cells were cultured using neural induction media supplemented with 3 μM CHIR99021 and 2 μM on X-ray-inactivated MEF feeders or Matrigel-coated plates. On days 6–10, induction factors were withdrawn, and PD173074 (0.2 μM) + DAPT (10 μM) was used for retinal ganglion cell induction. On days 10–15, the medium was replaced with N2, B27, 300 μg/mL cAMP (Sigma-Aldrich, St. Louis, MO, USA), 100 ng/mL SHH (C24 II), and 100 ng/mL FGF8b. Next, 10 ng/mL BDNF, 10 ng/mL GDNF, 10 ng/mL IGF-1, 1 ng/mL TGF-β, and 0.5 mM db-cAMP were added and the cells were cultured for 30 days. The culture medium for this stage was named DAn-modified Eagle’s medium (DAn-MEM), which was used for subsequent DAn cultures. The cells were collected and dopaminergic neuronal markers such as tyrosine hydroxylase (TH) and β3-Tubulin (TUJ1) were detected using immunofluorescence staining and RT-qPCR. TH antibody (CST-2791), TUJ1 (TU-20) antibody (CST-4466), and DAPI (Sigma-D9542) were used for immunofluorescence detection. The primers used for DAn-specific genes are listed in **Table 2**. As described in our previous report (21), we utilized electrophysiological analysis to confirm the identity of human DAn.

**Table 2:** Primers applied in the RT-qPCR.

Gene	Forward (5’ to 3’)	Reverse (5’ to 3’)
Homo-*GAPDH*	CGGAGTCAACGGATTTGGTC	GACAAGCTTCCCGTTCTCAG
Homo-*TUBB3*	GCCTCTTCTCACAAGTACGTGCCTCG	GGGCGAAGCCGGGCATGAACAAGTGCA
Homo-*TH*	GCCCTACCAAGACCAGACGTA	CGTGAGGCATAGCTCCTGAG
Homo-*FOXA2*	GGGAGCGGTGAAGATGGA	TCATGTTGCTCACGGAGGAGTA
Homo-*PITX3*	GTGCGGGTGTGGTTCAAGAA	AGCTGCCTTTGCATAGCTCG
Homo-*HO-1*	AAGACTGCGTTCCTGCTCAAC	AAAGCCCTACAGCAACTGTCG
Homo-*NQO1*	GAAGAGCACTGATCGTACTGGC	GGATACTGAAAGTTCGCAGGG
Homo-*MRP2*	AGTGAATGACATCTTCACGTTTG	CTTGCAAAGGAGATCAGCAA
Homo-*GPX2*	CTGGTGGTCCTTGGCTTC	TGTTCAGGATCTCCTCATTCTG
Homo-*Bcl2*	GCGACTCCTGATTCATTGGG	ACTTCCTCTGTGATGTTGTATTT
Homo-*Bax*	CATGGGCTGGACATTGGACT	GAGAGGAGGCCGTCCCAA
Rat-*GAPDH*	CCTCGTCTCATAGACAAGAT	GGGTAGAGTCATACTGGAA
Rat-*HO-1*	TGCACATCCGTGCAGAGAAT	CTGGGTTCTGCTTGTTTCGC
Rat-*NQO1*	AGGATGGGAGGTACTCGAATC	TGCTAGAGATGACTCGGAAGG
Rat-*MRP2*	GCCCCTCAAGCACTCTGAC	GCTTTGTGTCCCAGATGGACT
Rat-*GPX2*	GAGCTGCAATGTCGCTTTCC	TGGGTAAGACTAAAGGTGGGC

### Immunofluorescence

The cells were fixed using 4% (v/v) paraformaldehyde (Alfa Aesar, Haverhill, MA, USA), washed three times with phosphate-buffer saline (PBS) containing 0.2% v/v Tween (PBST; Thermo Fisher Scientific), and permeabilized using 0.15% v/v TritionX-100 (Sigma-Aldrich) in PBS for 1 h at 25°C. After gentle removal of PBST, the cells were incubated with primary antibodies (Nrf2, TH and TUJ1) in PBST overnight at 4°C. Subsequently, the cells were washed three times with PBST and stained with the secondary antibody for 1 h at 37°C. The cells were thrice washed in PBST, stained with DAPI, and viewed under a laser scanning confocal microscope (Carl Zeiss-710). To observe the ratio of DAn, the proportion of TH^+^/TUJ1^+^ double-positive cells among all cells was statistically analyzed. The quantitative statistics of fluorescence intensity and localization of the related proteins were performed as we previously reported (21).

### The establishment of a DAn oxidation model

Cultured DAn were treated with 100 μM H_2_O_2_ for 12 h according to a previously reported protocol (22). After treatment, the apoptosis and reactive oxygen species (ROS) levels in the cells were examined with flow cytometry. Immunofluorescence (IF) data showed that the percentage of TH/TUJ1 double-positive cells in H_2_O_2_-treated cells was decreased significantly, which is considered a model of oxidative damage of DAn.

### Experimental grouping

DAn culture were randomly divided into four groups: control (Ctrl), oxidative damage model (Model), model treated with blank serum (Blank Serum), and model treated with PB-II-treated serum group (PB-II Serum). Control cells were cultured under normal conditions (DAn-MEM), whereas the other cultures were treated separately. We added 10% rat serum to the Blank Serum group and 10% PB-II-treated medicated serum to the PB-II Serum group for 24 h. The following day, the three treatment groups (Blank Serum, PB-II Serum, and Model) were treated with 100 μM H_2_O_2_ for another 12 h. Finally, all cell samples were examined for apoptosis, DAn neuronal activity, ROS, and Nrf2 signaling pathway and associated gene expression.

### Flow cytometry analysis

Flow cytometry was used to analyze TH-positive cells, apoptosis, and ROS levels. Intracellular staining was performed to detect THs. The cells were digested, fixed with 4% paraformaldehyde, blocked with BSA, and incubated with anti-TH antibody (ab75875, 1/100 dilution) for 30 min at 22°C. The secondary antibody used was DyLight- 488 goat anti-rabbit IgG (H+L) (ab96899) at a 1/500 dilution for 30 min at 22°C. A total of >5,000 events were acquired. For apoptosis detection, the KEYGEN apoptosis kit (^#^KGA108–1) was used according to the manufacturer’s instructions. Annexin V-FITC/PI staining was performed using flow cytometry software (BD Biosciences, Franklin Lakes, NJ, USA). ROS were detected using the Reactive Oxygen Species kit (^#^KGT010–1) according to the manufacturer’s instructions. ROS detection was based on the fluorescent probe DCFH-DA. Intracellular ROS can oxidize non-colored DCFH to fluorescent DCF. Thus, flow cytometry can be used to detect ROS fluorescence intensity.

### Establishment of a reporter cell line and luciferase reporter gene assay

Based on prior research in our lab, anti-oxidant responsive element (ARE)-luciferase plasmid was constructed by inserting a 39-bp ARE-containing sequence from the promoter region of the human NAD(P)H quinone oxidoreductase 1 (*NQO1*) gene into the cloning site of the pGL4.22[luc2CP/Puro] plasmid. We transfected ARE-luciferase plasmid into MDA-MB-231 cells and used 1.5μg/mL puromycin to selected the stable reporter cell lines. For the dual luciferase reporter gene assay, MDA-MB-231 cells were co-transfected with ARE-luciferase plasmid and Renilla luciferase plasmid. The transfected cells were treated with H_2_O_2_ or PB-II for 24 h. Luciferase activity was monitored with Dual-Luciferase Reporter Assay (Promega, J3082, USA) according to manufacturer’s instructions.

### Animal experiment

Sprague-Dawley rats (male, 220–250 g) were purchased from Guangdong Medical Laboratory Animal Center (Guangzhou, China). All animal experiments were approved by the Animal Review Board of Guangdong Provincial Hospital of Chinese Medicine (approval number: 2018009). Rats were housed under constant temperature (20–22°C) and a 12 h light-dark cycle, with free access to food and water. After one week of acclimation to the environment, the APO-induced rotation test (see section “APO-induced rotation test”) was performed before being used for modeling (23). Rats without rotation were used for stereotaxic injections.

Rats were separated into four groups (*n* = 5): control, sham, model, and PB-II. The rats in the control group were intragastrically administered with distilled water. The sham group was injected with vehicle and distilled water. The model group was injected with 6-OHDA (see the injection procedure) and distilled water. The PB-II group was injected with 6-OHDA and then administered with PB-II (32 g/kg). All intragastric treatments were continued for four weeks after modeling.

The stereotaxic injection procedure was as following: After being anesthesized with 3% pentobarbital sodium (50 mg/kg, i. p.), the rats were placed in a stereotaxic apparatus. 6-OHDA (3 µL, 5 mg/mL; 0.02% of ascorbic acid in normal saline) was injected unilaterally into each injection site of the left striatum (coordinates from bregma: site 1: antero-posterior: 1.0 mm, medio-lateral: 4.4 mm, dorso-ventral: -4.5 to -6.5 mm; site 2: antero-posterior: -1.2 mm, medio-lateral: 2.2 mm, dorso-ventral: -4.0 to -6.0 mm). The injection rate was 1 µL/min. Rats in the sham group were injected with 0.02% ascorbic acid in normal saline.

### APO-induced rotation test

An APO-induced rotation test was conducted to evaluate motor function at weeks two, four, six, and eight post-6-OHDA injection. The APO was dissolved in normal saline containing 0.02% ascorbic acid. Rats were subcutaneously injected with 0.5 mg/kg APO (24). After 5 min, the number of contralateral rotations was recorded for 30 min.

### Immunohistochemistry

The rats were anesthetized with 3% pentobarbital sodium (50 mg/kg, i. p.) and perfused with normal saline and 4% paraformaldehyde. Brains were isolated and fixed in 4% paraformaldehyde for 24 h. Brain tissues were dehydrated and embedded in paraffin. The coronal brain sections were prepared using a microtome. Sections were dewaxed, hydrated, and heated in citric acid buffer (pH 6.0) for 20 min. After incubation with 3% hydrogen peroxide for 15 min, the sections were washed with PBS and incubated with blocking solution for 30 min at 37°C. The primary antibody (TH 1:1000) was added to the sections and incubated overnight at 4°C. The sections were then washed with PBS and incubated with HRP-conjugated secondary antibody for 30 min at 37°C. Another three-wash step involved a DAB reaction for 5 min. Finally, sections were washed with water and dehydrated using an ethanol gradient. The images were captured using a BX61 microscope (Olympus, Tokyo, Japan).

### ELISA

The following human DAn ELISA kits were used: Monocyte Chemoattractant Protein-1 (MCP-1) (EHC113.96, Neobioscience, Shenzhen, China), tumor necrosis factor-α (TNF-α) (CSB-E09315h, Cusabio, Wuhan, China), interleukin 6 (IL-6) (CSB-E04638h, Cusabio, Wuhan, China), and interleukin 10 (IL-10) (CSB-E04593h, Cusabio, Wuhan, China). The following rat nigrostriatal region tissue ELISA kits were used: MCP-1 (CSB-E07429r, Cusabio, Wuhan, China), TNF-α (ERC102a.96, Neobioscience, Shenzhen, China), IL-6 (ERC003.96, Neobioscience, Shenzhen, China), and IL-10 (CSB-E04595r, Cusabio, Wuhan, China). Analyses were performed according to the manufacturer’s instructions.

### Transcriptome analysis

Total RNA from rat midbrains was extracted with TRIzol^®^ Reagent (Invitrogen, Carlsbad, CA, USA) according to the manufacturer’s instructions and then enriched and purified using Oligo(dT) magnetic beads (Thermo Fisher Scientific) through direct targeted hybridization. mRNA-seq libraries were prepared and constructed using the VAHTS Stranded mRNA-seq Library Prep Kit (Vazyme Biotech, Jiangsu, China), following the manufacturer’s instructions. The raw sequence data reported in this paper have been deposited in the Genome Sequence Archive (Genomics, Proteomics & Bioinformatics 2021) at the National Genomics Data Center (Nucleic Acids Res 2022), China National Center for Bioinformation/Beijing Institute of Genomics, Chinese Academy of Sciences (GSA: CRA008871) and are publicly accessible at https://ngdc.cncb.ac.cn/gsa. Qualified libraries were sequenced using an Illumina NovaSeq 6000 system in a paired-end format. Reads were mapped to the reference genome (Rnor_6.0.104, ENSEMBL) using Hisat2 and feature counts were used to count the reads. Fragments per kilobase million were calculated after normalization to the trimmed mean of M values. The edgeR package was used to analyze significantly differentially expressed genes. Gene set enrichment analysis (GSEA) was performed using the pre-ranked method in GSEA Java implementation (25). The WIKI pathway database (MsigDB, http://software.broadinstitute.org/gsea/) was used for gene annotation, and a *P*-value <0.05 was considered statistically significant.

### Nrf2-antioxidant response element signal detection

Western blotting was performed to detect the Nrf2 protein in cells and the midbrains of rats after various treatments. ImageJ software was used to analyze the protein gray values. RT-qPCR was used to detect the mRNAs of Nrf2 downstream genes, such as heme oxygenase-1 (*HO-1*), *NQO1*, multidrug resistance-associated Protein 2 (*MRP2*), and glutathione peroxidase 2 (*GPX2*). The primer sequences are shown in **Table 2**.

### Quantification and statistical analysis

Data are represented as the mean ± SEM unless otherwise indicated, and Student’s *t*-test was used to compare two groups. One-way ANOVA was used to compare multiple groups. GraphPad Prism 5 software was used for statistical analyses. Differences between two groups were considered significant when the *P*-value was <0.05.

## Results

### Quality control test of medicated serum

**Figure 1:**
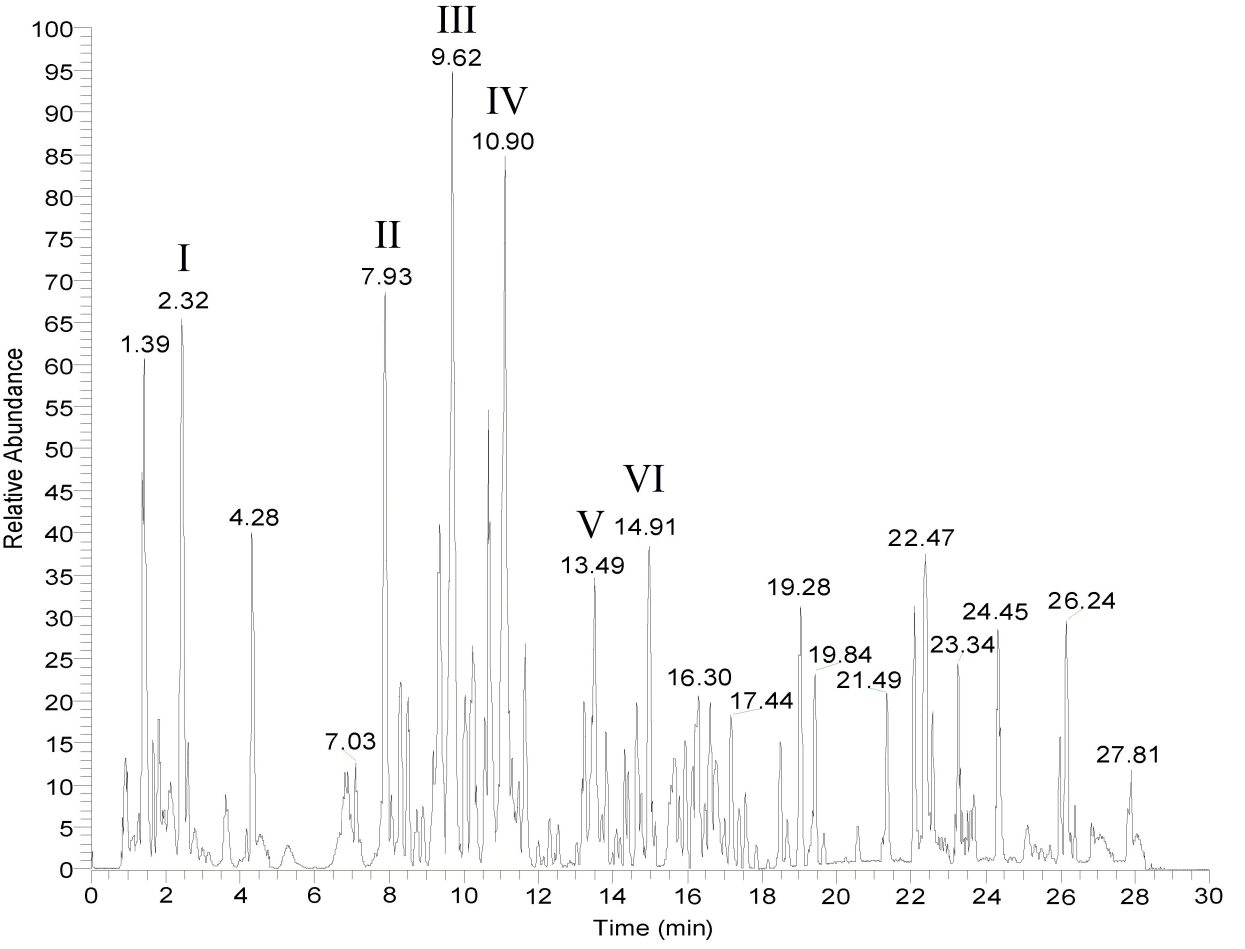
LC-MS spectrum for PB-II. The mass spectrum of PB-II was obtained using LC-MS in drug-containing serum. (I) Citric acid; (II) Hypaconitine; (III) Stibene glucoside; (IV) Liquiritin; (V) Paeoniflorin; (VI) Ginsenoside Rg1.

LC-MS was used to analyze the active ingredients in the medicated serum and the six main compounds in the recipe, citric acid, hypaconitine, stilbene glucoside, glycyrrhizin, paeoniflorin, and Ginsenoside Rg1, were analyzed (**Figure 1**). Identification and simultaneous detection were performed to provide a reference for the quality control of PB-II. A detailed map is shown in **Figure 1**.

### Generation of hiPSC and their differentiation into DAn

**Figure 2:**
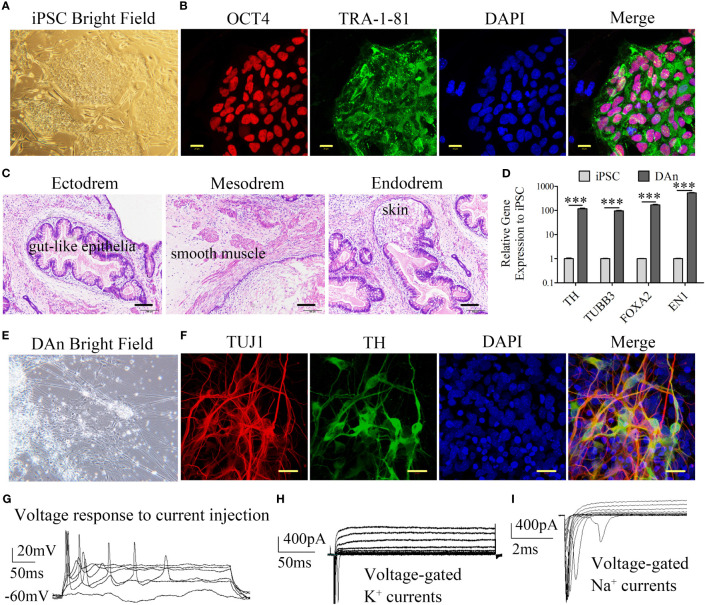
Generation of iPSC and differentiation into the dopaminergic neuron. **(A, B)** Representative colonies of passage-20 iPSC stained positive for the pluripotency-associated markers OCT4 and TRA-1–81 (scale bars, 20 μm). **(C)** Teratomas derived from iPSC have the pluripotency of three germ layers. The sections with hematoxylin and eosin staining showed the germ layers of ectoderm, mesoderm, and endoderm differentiation (scale bars, 100 μm). **(D)** RT-qPCR showed that the iPSC-DAn expression to the iPSC lines is approximately 100 times that of neuron relative genes such *TH*, *TUBB3*, *FOXA2*, and *EN1* (*n*=3, ^***^ *P*<0.001). **(E)** Differentiated cells have the morphology of DAn. **(F)** Immunofluorescence staining of neuronal cultures derived from iPSC for neuron-specific TUJ1 (red), the DA marker TH (green), and nuclear DAPI (blue) (scale bars, 20 μm). **(G)** DAn fired evoked action potential and had voltage-gated K^+^ and Na^+^ currents **(H, I)** shown by cell patch clamp electrophysiology.

An iPSC line was reprogrammed from healthy human skin fibroblasts as previously described (20). iPS cells were cultured in ESC medium containing DMEM/F12 (11330; Gibco, Billings, MT, USA), KnockOut™ Serum Replacement (Gibco), MEM non-essential amino acids (Gibco), L-glutamine (Gibco), 55 mM β-mercaptoethanol (Gibco), and 20 ng bFGF (Gibco) on feeders made from CF1 mouse embryos and subjected to radioactive irradiation at a dose of 30 Gy. Colonies with the hESC morphology were observed between days 25 and 45. These cells were selected and expanded under hESC culture conditions. The pluripotency of the hiPSC line was confirmed by colony morphology, expression of the pluripotency markers OCT4 and TRA-1–81, and the formation of teratomas in NSG mice (**Figures 2A–C**). These data confirm the pluripotency of hiPSCs.

Using a previously described protocol (26), hiPSCs were differentiated into DAn. The presence of DAn in differentiating cultures was confirmed using DAn-specific markers. DAn-specific genes such as *TH*, *TUBB3* (*TUJ1*), *FOXA2*, and Engrailed 1 (*EN1*) were significantly upregulated in iPSC-derived DAn (**Figure 2D**). In addition, cell morphology confirmed the morphological characteristics of DAn (**Figure 2E**). IF analysis demonstrated that approximately 60% of iPSC-derived cells expressed the neuron-specific marker TUJ1 and approximately 40% of iPSC-derived neurons were TH^+^/TUJ1^+^, confirming the presence of hiPSC-derived DAn (**Figure 2F**). Electrophysiological assays showed that the DAn possessed electrophysiological phenomena specific to human dopaminergic neurons (**Figures 2G–I**).

### PB-II protects DAn from oxidative damage induced by H_2_O_2_

**Figure 3:**
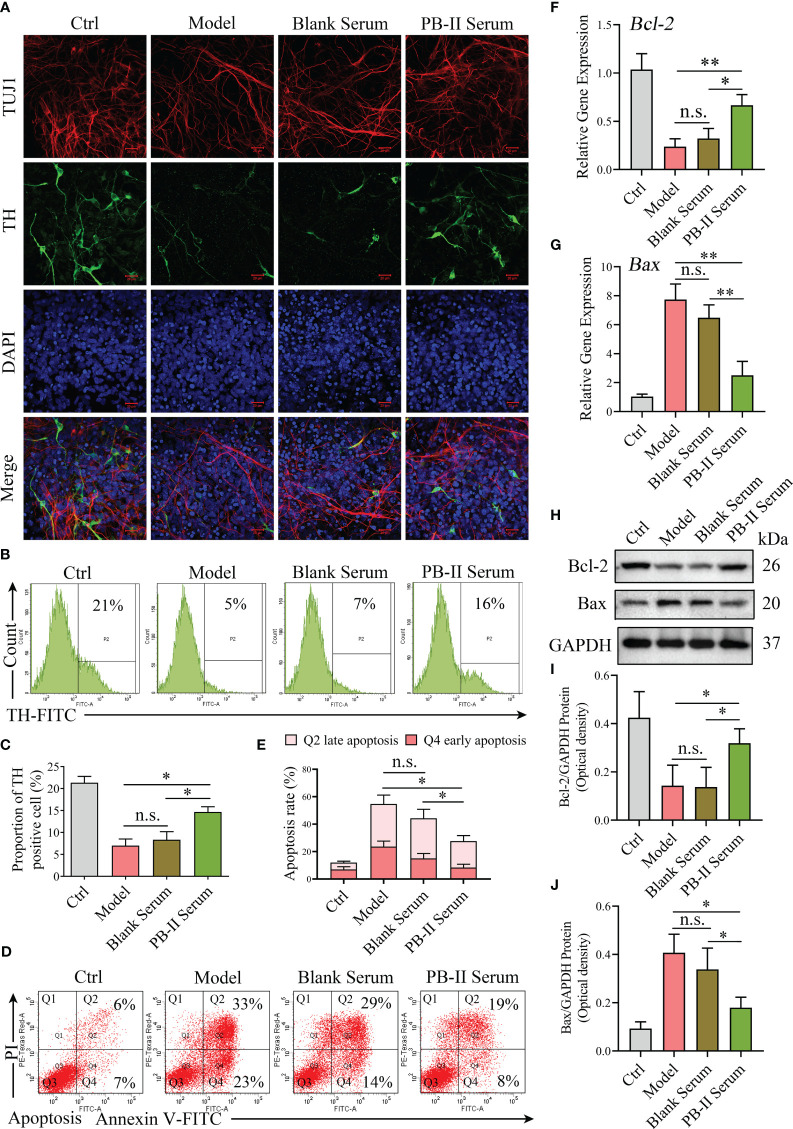
PB-II protects the neuronal cell with activated neuronal expressions. **(A)** Immunofluorescence data showed more TUJ1 and TH neuronal staining in PB-II Serum sample than that in the Model and Blank Serum samples (scale bars, 20 μm). **(B, C)** Flow cytometry analysis of the proportion of TH^+^ cells in each experimental group, compared with that of the Model and Blank Serum groups, the PB-II Serum group had a higher percentage of TH^+^ cells, with statistically significant differences (*n* = 3, ^*^ *P*<0.05; *n.s*., no significance). **(D, E)** Flow cytometry detected the proportion of apoptosis in each experimental group. Compared with that of the Model and Blank Serum groups, the apoptosis in the PB-II Serum group decreased significantly (*n* = 3, ^*^ *P*<0.05; *n.s*., no significance). **(F, G)** RT-qPCR data of *Bcl-2* and *Bax* mRNA expression in DAn of each experimental group (*n* = 3, ^*^ *P*<0.05; ^**^ *P*<0.01; n.s., no significance). **(H–J)** Immunoblotting data of Bcl-2 and Bax protein expression in DAn of each experimental group (*n* = 3, ^*^ *P*<0.05; n.s., no significance).

Immunofluorescence data showed more TUJ1^+^/TH^+^ neurons in the PB-II Serum samples than in the Model samples prepared using H_2_O_2_ damage and Blank Serum samples (**Figure 3A**). While TUJ1^+^/TH^+^ neurons decreased in the Model group after treatment with H_2_O_2_, PB-II Serum significantly increased the number of TUJ1^+^/TH^+^ neurons, indicating that PB-II Serum protected TUJ1^+^/TH^+^ neurons from oxidative stress (**Figure 3A**). In support of this conclusion, flow cytometric analysis indicated that the percentage of TH^+^ cells in the PB-II Serum group was significantly higher than that in the Model or Blank Serum groups (**Figures 3B, C**). The percentage of apoptotic cells was analyzed in each experimental group, indicating that PB-II Serum protected DAn from apoptosis after H_2_O_2_ treatment (**Figures 3D, E**). Furthermore, when compared to that in the Ctrl sample, the expression of *Bcl-2* mRNA and protein in Model was significantly decreased, while the expression of *Bax* mRNA and protein was significantly increased (**Figures 3F-J**), indicating that apoptosis of DAn was significantly increased after oxidative stress. PB-II could significantly reduce the apoptosis of DAn after oxidative stress (**Figures 3D–J**).

### PB-II activates the Nrf2/ARE signaling pathway and reduces cellular ROS

**Figure 4:**
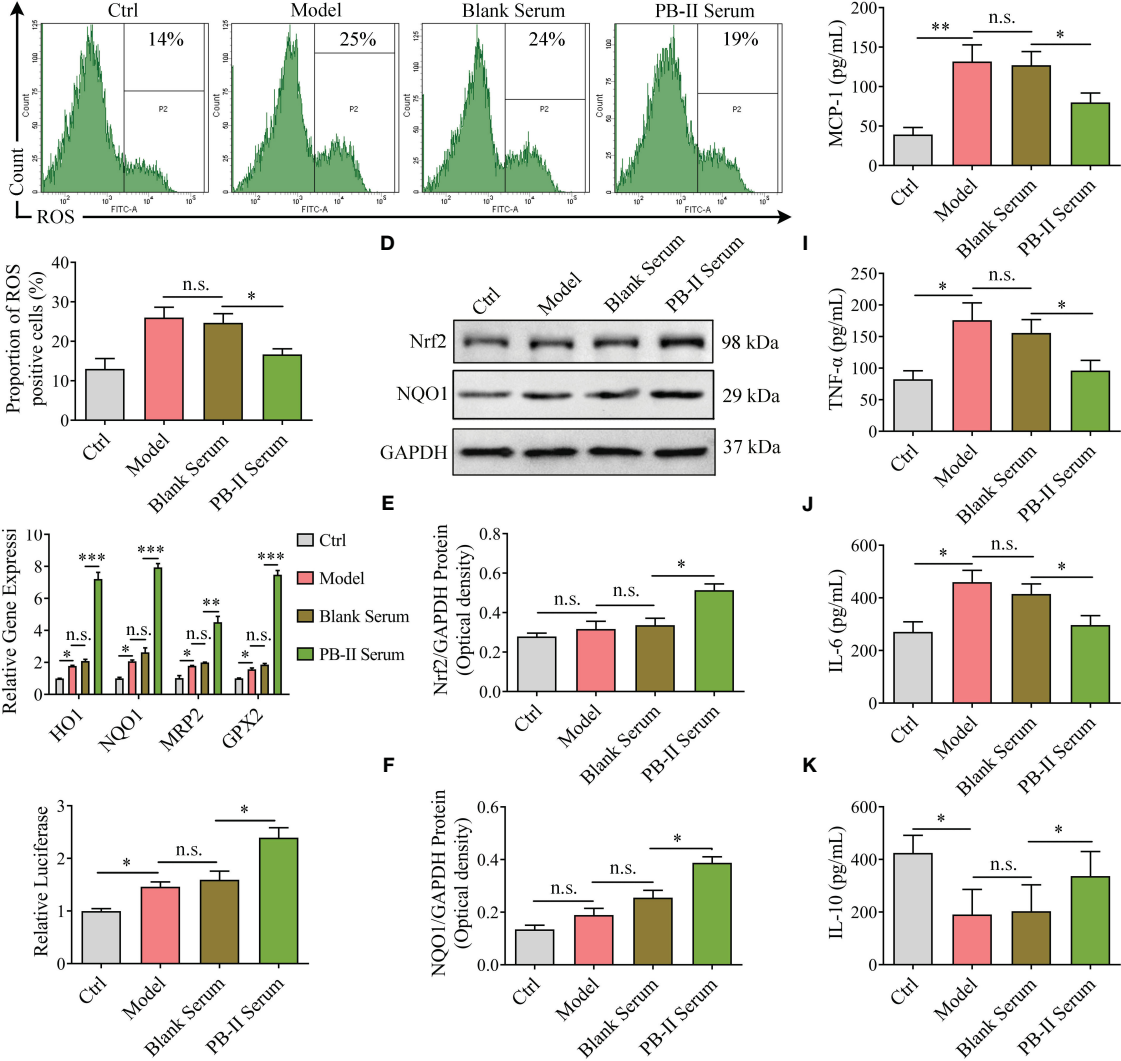
Nrf2 pathway genes are activated in medicated cells and reduce ROS and inflammation. **(A, B)** The higher ROS level activated under H_2_O_2_ stress was inhibited by PB-II Serum (*n* = 3, ^*^ *P*<0.05; *n.s*., no significance). **(C)** The Nrf2 signaling pathway downstream genes, *HO-1*, *NQO1*, *MRP2*, and *GPX2*, were found in higher levels in PB-II Serum samples than in Model or Blank Serum samples (*n* = 3, ^*^ *P*<0.05, ^**^ *P*<0.01, ^***^ *P*<0.001; *n.s*., no significance). **(D–F)** Western blotting showed that the Nrf2 and NQO1 proteins in PB-II Serum samples are significantly more increased than those in the other samples (*n* = 3, ^*^ *P*<0.05; *n.s*., no significance). **(G)** Luciferase activity was tested with corresponding Kit (*n* = 3, ^*^ *P*<0.05; *n.s*., no significance). **(H–K)** ELISA data showed that the pro-inflammatory factors, MCP-1, TNF-α, and IL-6 in the model group increased, while the anti-inflammatory factor IL-10 decreased. The intervention of PB-II could reduce pro-inflammatory factors and increase anti-inflammatory factors (*n* = 3, ^*^ *P*<0.05, ^**^ *P*<0.01; *n.s*., no significance).

To explore the mechanism by which PB-II protects DAn from oxidative stress and inflammation, the ROS levels and inflammatory cytokines in hiPSC-derived neuronal cultures were examined after various treatments. Although ROS levels were similar between the Model and Blank Serum treatment groups, PB-II Serum significantly decreased cellular ROS levels in DAn after oxidative stress, supporting the role of PB-II in reducing oxidative stress in DAn (**Figures 4A, B**; **Supplementary Figures S1E, F**). Collectively, these findings support the hypothesis that PB-II protects DAn from oxidative stress by reducing cellular ROS levels.

As previously reported, ROS trigger the redox system by activating Nrf2 partly by promoting its nuclear translocation, leading to the increased expression of its downstream genes, such as *HO-1*, *NQO1*, *MRP2*, and *GPX2*. After treatment with H_2_O_2_ (100 μM) for 12 h, Nrf2 protein levels and downstream gene expression were similar to those of the Ctrl group (**Figures 4C–F**; **Supplementary Figures S1A–D**). However, PB-II Serum treatment significantly increased the expression of Nrf2 protein and its downstream genes, such as *NQO1* (**Figures 4C–F**; **Supplementary Figures S1A–D**). PB-II Serum significantly induced Nrf2 nuclear translocation in oxidative stress DAn by H_2_O_2_ intervention with effects comparable to artemisitene (ATT), a known Nrf2 activator (**Supplementary Figures S2A–D**). Employing the MDA-MB-231 cells stably transfected with an ARE-luciferase reporter as a screening platform (27), we identified that PB-II could induce the expression of the ARE-dependent luciferase gene (**Figure 4G**). ELISA detection showed that H_2_O_2_ treatment significantly increased the expression of pro-inflammatory factors in DAn cells, such as MCP-1, TNF-α and IL-6, while inhibiting the expression of anti-inflammatory factors, IL-10. The treatment of PB-II could significantly reduce the inflammatory response of the Model group (**Figures 4H–K**; **Supplementary Figures S1G-J**). Therefore, PB-II activates the Nrf2/ARE signaling pathway to protect DAn from oxidative stress and inflammation.

### PB-II improves the symptoms of PD rats by activating the Nrf2/ARE signaling pathway

**Figure 5:**
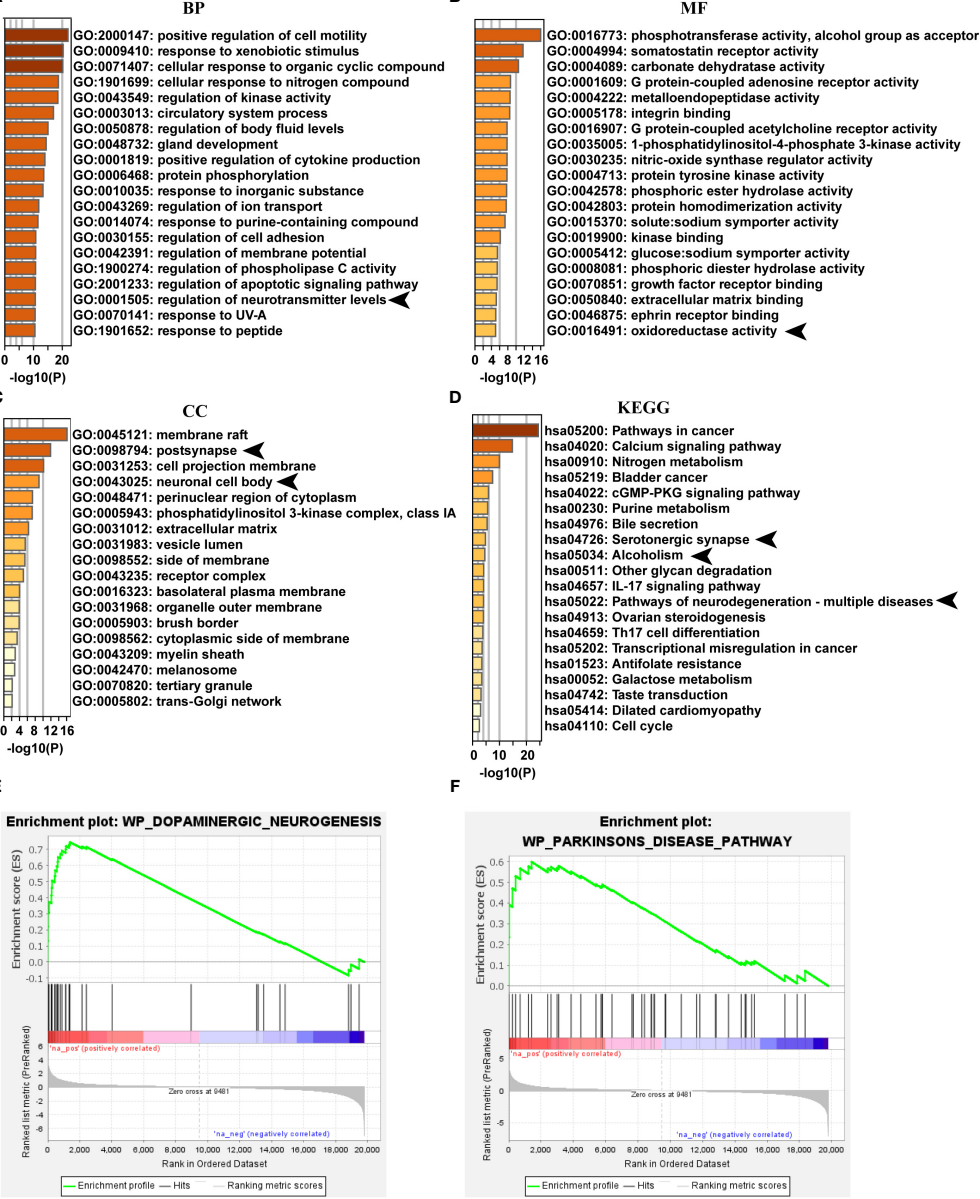
PB-II targets PD pathway. **(A)** Top 20 clusters of GO biological process enrichment. **(B)** Top 20 clusters of GO molecular functions enrichment. **(C)** Top 20 clusters of GO cellular components enrichment. **(D)** Top 20 clusters of KEGG enrichment. **(E)** GSEA analysis of dopaminergic neurogenesis pathway between PB-II and model groups (NES = 2.07, *P*<0.01). **(F)** GSEA analysis of Parkinson’s disease pathway between PB-II and model groups (NES = 1.78, *P* < 0.01). CC, cellular components; BP, biological processes; MF, molecular functions. Arrows indicate the neuronal signaling associated with GO enrichment items and KEGG pathways.

Network pharmacology analysis of the main identified compounds of PB-II-treated serum showed that several neuronal signalings were involved in the GO functions, such as “regulation of neurotransmitter levels” (**Figure 5A**), “oxidoreductase activity” (**Figure 5B**), “postsynapse”, and “neuronal cell body” (**Figure 5C**). The top 20 KEGG pathways of PB-II-treated serum also included neuron-associated pathways, such as “serotonergic synapse”, “alcoholism”, and “pathways of neurodegeneration-multiple diseases” (**Figure 5D**). Dopaminergic neuronal signaling has been implicated in neurotransmission, alcoholism, and neurodegenerative diseases. Transcriptome analysis was performed to better understand the mechanism of action of PB-II in PD. GSEA analysis showed that leading-edge genes associated with “dopaminergic neurogenesis” and “Parkinson’s disease” pathways were highly expressed, following PB-II treatment as compared to the model group (**Figures 5E, F**), suggesting that PB-II regulates dopaminergic neuronal signaling and the PD pathway.

**Figure 6:**
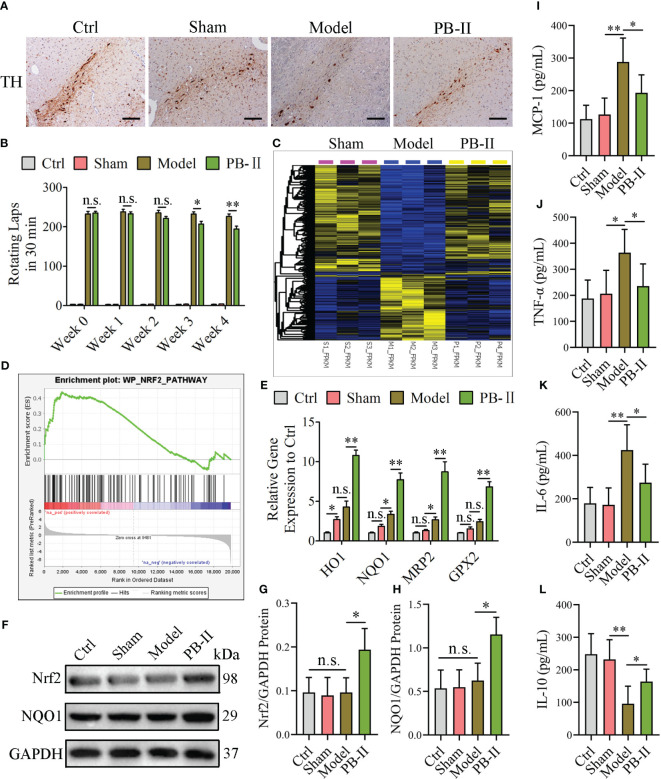
PB-II protects PD rat DAn by activating the Nrf2/ARE signaling pathway. **(A)** Immunohistochemical results showed that the TH-positive neurons in the substantia nigra of the PB-II group were significantly increased compared with the sham and model groups (scale bars, 120 μm). **(B)** The number of rotations of the rats in each experimental group within 30 min was calculated. From the third week, the number of rotations of the PD-rats in the PB-II group decreased compared with the model group (*n* = 5, ^*^ *P*<0.05; n.s., no significance) and reached a very significant difference in the fourth week (*n* = 5, ^**^ *P*<0.01; n.s., no significance). **(C)** Heat map of differentially expressed genes among the sham (pink), model (blue), and PB-II groups (yellow) obtained from the transcriptome analysis (*n* = 3). **(D)** GSEA analysis of the NRF2 pathway between the PB-II and model groups showed that the NRF2 pathway was highly expressed in the PB-II group (NES = 1.53, *P* < 0.01). **(E)** RT-qPCR results showed that the expression of Nrf2 downstream genes in the substantia nigra tissues of rats in the PB-II group was significantly increased, suggesting the activation of the Nrf2 signaling pathway of PB-II on DAn cells under 6-OHDA toxicity (*n* = 3, ^*^ *P*<0.05, ^**^ *P*<0.01; n.s., no significance). **(F–H)** Western blotting detection results showed that Nrf2 and NQO1 protein expression in cells of the substantia nigra region of the midbrain in the PB-II group of rats increased significantly (*n* = 3, ^*^ *P*<0.05; n.s., no significance). **(I–L)** ELISA assay results showed that the MCP-1, TNF-α, and IL-6 increased in the model group, while the IL-10 decreased. PB-II treatment could reduce pro-inflammatory factors and increase IL-10 (*n* = 3, ^*^ *P*<0.05, ^**^ *P*<0.01; n.s., no significance).

To further validate that PB-II can activate the Nrf2/ARE signaling pathway to protect DAn from oxidative stress and inflammation, the effects of PB-II in PD rat models were tested by injecting 6-OHDA into the striatum of rats to induce midbrain DAn death (model group). The sham operation group was injected with an equal volume of normal saline (sham group). The PB-II group was administered 32 g/kg PB-II via gavage. During the four-week treatment course, the behavioral symptoms of the PD rats were measured weekly. Spinal behavior in PD rats was induced by subcutaneous injection of APO into the back of the neck, and the number of rotations was recorded within 30 min. During the initial stage of treatment (week 0), the rats in the control and sham groups showed no symptoms of *in situ* circles. However, the model and PB-II groups showed severe rotationary behavior, with more than 210 rotations in 30 min, and there was no significant difference between the two groups. During the third week of treatment, the number of rotations was significantly reduced in the PB-II group (**Figure 6B**). After four weeks of treatment, the rats were euthanized and tissues of the nigrostriatal region were obtained. The number of TH^+^ neurons in the substantia nigra of rats in the PB-II group was significantly higher than those in the sham and model groups (**Figure 6A**). In addition, transcriptome analysis showed that overall gene expression in the PB-II group was similar to that in the sham group (**Figure 6C**). GSEA revealed that the target genes associated with the Nrf2 pathway were highly expressed after PB-II treatment compared to those in the model group (**Figure 6D**). Furthermore, the expression of *HO-1*, *NQO1*, *MRP2*, and *GPX2* in the midbrain of the PB-II group was also significantly increased, indicating the activation of the Nrf2/ARE signaling pathway by PB-II (**Figure 6E**). The Nrf2 and NQO1 protein levels in the midbrain DAn of rats in the PB-II group were significantly higher than those in the sham and model groups (**Figures 6F–H**). Meanwhile, the inflammatory response in the model group was intensified, while PB-II treatment significantly reduced the inflammation (**Figures 6I–L**). These data confirm that PB-II activates the Nrf2/ARE signaling pathway in the midbrain of PD rats by activating Nrf2 to reduce oxidative stress and inflammation in the DAn, thus protecting the DAn from oxidative stress and inflammation-induced apoptosis.

## Discussion

Oxidative stress is believed to cause the death of DAn (28–30), and the activation of the endogenous antioxidant system may protect cells from oxidative damage (30). Multiple studies in various organs have confirmed that the Nrf2-ARE pathway acts as an endogenous antioxidant pathway that antagonizes oxidative stress injury (31). In cells of the central nervous system, such as DAn, astrocytes, and microglia, Nrf2 maintains redox balance through the upregulation of antioxidant genes (32). Activation of the Nrf2 signaling pathway can also reduce inflammatory responses (33). Previous research has verified that Nrf2 is mostly translocated to the nucleus of DAn in the substantia nigra of patients with PD, whereas it is present in the cytoplasm of a matched normal control group of the same age (17, 34, 35). Studies have also demonstrated that Nrf2 overexpression can reduce the damage caused by 6-OHDA in DAn (28, 36). Under physiological conditions, Nrf2 protein expression is low in cells and mainly in the cytoplasm where it interacts with Kelch-like ECH-associated protein-1 (Keap1) (37, 38). In response to oxidative stress, Nrf2 is dissociated from KEAP1 and translocated into nucleus to regulate ARE, inducing the expression of downstream target genes such as *HO-1* and *NQO1*, and thus enhancing the detoxification and antioxidant ability of cells (39–41). *In vitro* experiments have also shown that the upregulation of HO-1 and NQO1 can protect cells against oxidative damage caused by glutamic acid, hydrogen peroxide, and amyloid-beta proteins (42–44).

Activation of the Nrf2 signaling pathway reduces the inflammation via multiple pathways, such as the inhibition of the nuclear factor kappa-B (NF-κB) (45) or PI3K/Akt (46) signaling pathways. In addition, a reduction in ROS would significantly reduce inflammation. In this context, ROS could activate NF-κB signal pathway (47), promoting inflammatory response and inducing α-synuclein aggregation in PD. In this context, ROS activates microglia to secrete several pro-inflammatory cytokines, such as TNF-α, IL-6, and MCP-1 (48). The inflammatory factors can also induce the expression of major histocompatibility complex (MHC) class II, which are associated with neuronal damage in PD patients.

Because traditional Chinese medicine (TCM) with herb components is delivered through the digestive system instead of intravenous injection, it is known that the digested and metabolized materials absorbed into the blood after digestive processes are the functional elements of TCM. Therefore, it is routine to use TCM-treated serum instead of TCM itself in functional studies of TCM. Our experiments show that PB-II-treated serum effectively reduced ROS levels and inflammation in an oxidative model of DAn, protecting DAn from apoptotic cell death. These results suggest that the Nrf2/ARE pathway-mediated antioxidant and anti-inflammatory mechanism may play a role in the effective treatment of neurodegenerative diseases. In support of this notion, our results show that PB-II plays a protective role against oxidative stress and inflammation in neurons by inducing the nuclear translocation and phosphorylation of Nrf2 as well as the expression of Nrf2 target genes.

PB-II contains 14 traditional berbs and its therapeutic efficacies for treating PD has been confirmed by many years of clinical practice (8–11). Furthermore, several studies have reported its protective effects on midbrain DAn against 6-OHDA toxicity in the substantia nigra of rat PD models (12–14). These studies further show the effects of PB-II in reducing apoptosis of DAn in the PD rat model, promoting cell regeneration, and improving PD symptoms in rats. However, the mechanisms underlying the protective roles of PB-II has been unclear. Our study provides a underlying mechanism by reducing the oxidative stress and inflammation through activation of Nrf2 pathway. Importantly, this study further confirms the powerful application of hiPSC derived neural cells in mechanistic studies of the complex TCM.

## Conclusions

PB-II activates the Nrf2 signaling pathway in DAn after oxidative stress, increasing the expression of antioxidant target genes of Nrf2, thereby improving the antioxidant capacity and survival of neurons by reducing ROS and inflammation. These findings explain the therapeutic efficacy of PB-II in the treatment of PD.

## Data availability statement

The raw transcriptome sequence data presented in the study are deposited in the Genome Sequence Archive (https://ngdc.cncb.ac.cn/gsa), accession number CRA008871. 

## Ethics statement

The studies involving humans were approved by the Institutional Review Board and TCM Review Board for Ethics of Guangdong Provincial Hospital. The studies were conducted in accordance with the local legislation and institutional requirements. The participants provided their written informed consent to participate in this study. The animal study was approved by the Animal Review Board of Guangdong Provincial Hospital of Chinese Medicine. The study was conducted in accordance with the local legislation and institutional requirements.

## Author contributions

SW: Conceptualization, Data curation, Formal analysis, Funding acquisition, Investigation, Methodology, Project administration, Resources, Validation, Visualization, Writing – original draft, Writing – review & editing, Software, Supervision. CR: Conceptualization, Data curation, Formal analysis, Investigation, Methodology, Software, Validation, Writing – review & editing, Resources, Visualization. RL: Data curation, Formal analysis, Investigation, Methodology, Software, Validation, Visualization, Writing – review & editing, Conceptualization. KJ: Conceptualization, Data curation, Formal analysis, Investigation, Methodology, Software, Validation, Visualization, Writing – review & editing. TL: Conceptualization, Data curation, Formal analysis, Investigation, Methodology, Project administration, Software, Supervision, Validation, Writing – review & editing. WC: Data curation, Investigation, Methodology, Software, Supervision, Writing – review & editing, Visualization. WM: Conceptualization, Data curation, Formal analysis, Funding acquisition, Investigation, Project administration, Resources, Supervision, Visualization, Writing – review & editing. YX: Conceptualization, Data curation, Formal analysis, Funding acquisition, Investigation, Methodology, Project administration, Resources, Software, Supervision, Validation, Visualization, Writing – original draft, Writing – review & editing.
