# Supplementary material for: Chinese medicine PaBing-II protects human iPSC-derived dopaminergic neurons from oxidative stress
Source: Front Immunol. 2024 Aug 2;15:1410784. doi: 10.3389/fimmu.2024.1410784 (PMC11327085; doi:10.3389/fimmu.2024.1410784)
Supplement: Supplementary file 1 [file DataSheet_1.docx]

**Chinese medicine PaBing-II protects human iPSC-derived dopaminergic neurons from oxidative stress**

# Shouhai Wu, Cuiping Rong, Ruishan Lin, Kaiyuan Ji, Tongxiang Lin, Weimin Chen, Wei Mao and Yang Xu


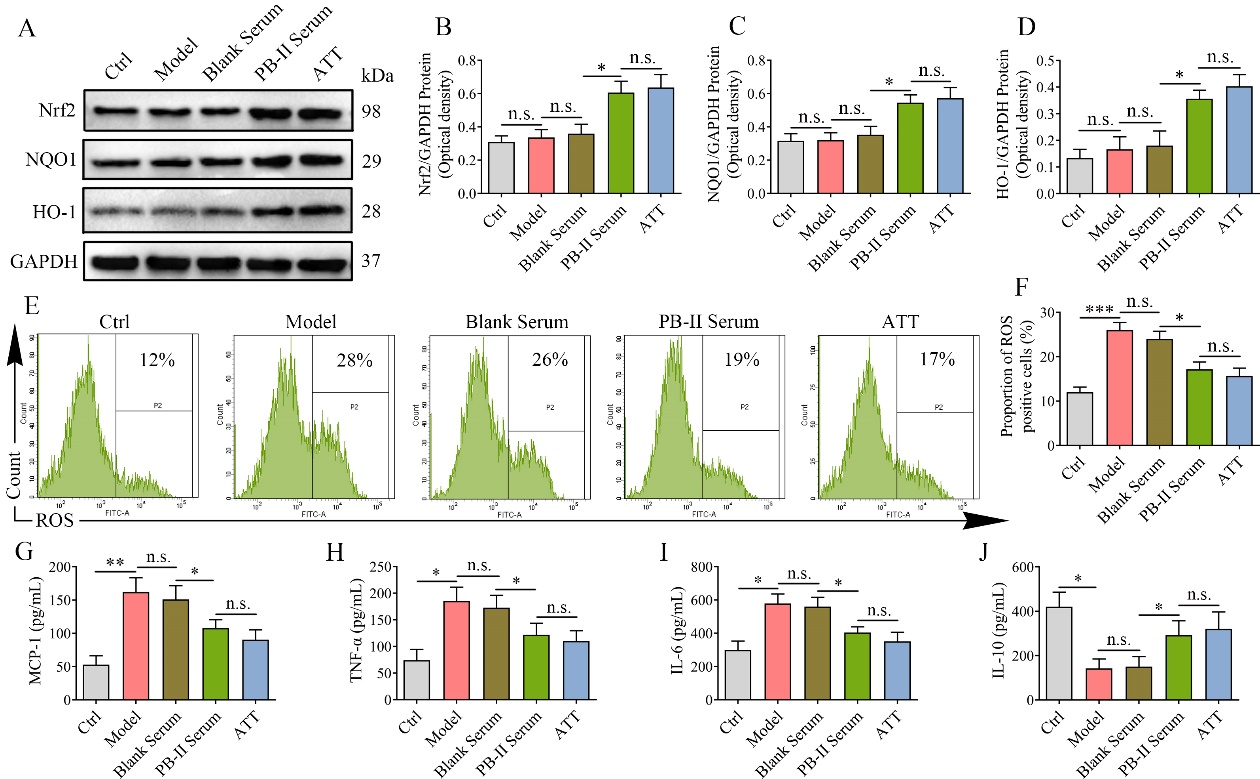


**Figure S1. PB-II and ATT activation of the Nrf2 signaling pathway. (A)** Western blotting showed that the Nrf2, NQO1 and HO-1 proteins in PB-II Serum and ATT samples are significantly more increased than those in the other samples. **(B-D)** Quantitative data on the expression of Nrf2 **(B)**, NQO1 **(C)**, and HO-1 **(D)** proteins (*n* = 3, **P*<0.05; n.s., no significance). **(E, F)** The higher ROS level activated under H_2_O_2_ stress was inhibited by PB-II Serum and ATT (*n* = 3, **P*<0.05, ****P*<0.001; n.s., no significance). **(G-J)** ELISA data showed that the pro-inflammatory factors, MCP-1, TNF-α, and IL-6 in the model group increased, while the anti-inflammatory factor IL-10 decreased. The intervention of PB-II and ATT could reduce pro-inflammatory factors and increase anti-inflammatory factors (*n* = 3, **P*<0.05, ***P*<0.01; n.s., no significance).


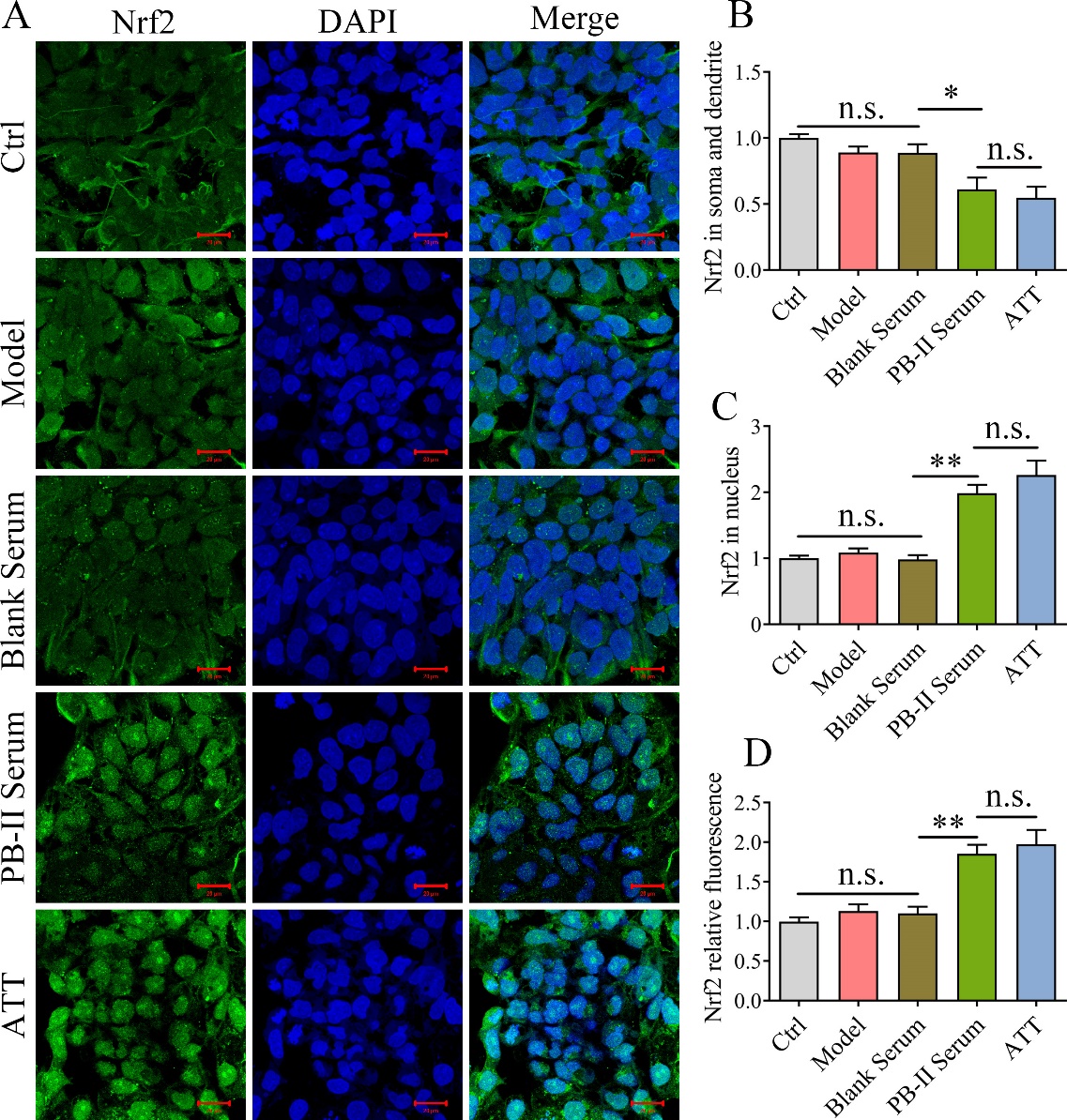


**Figure S2.** **PB-II induces Nrf2 nuclear translocation** **in oxidative stress DAn**. **(A)** Immunofluorescence data showed that PB-II and ATT can induce Nrf2 (green) nuclear transfer (Scale bars, 20 μm). **(B, C)** The quantitative data of the expression of Nrf2 in the cytoplasm soma **(B)** and nucleus **(C)**. **(D)** Quantitative data of fluorescence intensity of Nrf2. (*n* = 3, **P*<0.05, ***P*<0.01; n.s., no significance).
