# Supplementary material for: Chinese medicine PaBing-II protects human iPSC-derived dopaminergic neurons from oxidative stress
Source: Front Immunol. 2024 Aug 2;15:1410784. doi: 10.3389/fimmu.2024.1410784 (PMC11327085; doi:10.3389/fimmu.2024.1410784)
Supplement: Supplementary file 2 [file DataSheet_2.pdf]

Supporting Data to

Chinese medicine PaBing-II protects human iPSC-derived dopaminergic neurons from oxidative stress

Shouhai Wu[1,2,†,*], Cuiping Rong[1,3,†], Ruishan Lin[4,†], Kaiyuan Ji[5], Tongxiang Lin[1,6], Weimin Chen[1], Wei Mao[1,2,*] and Yang Xu[7,*]

The raw sequence data reported in this paper have been deposited in the Genome Sequence Archive (Genomics, Proteomics & Bioinformatics 2021) at the National Genomics Data Center (Nucleic Acids Res 2022), China National Center for Bioinformation/Beijing Institute of Genomics, Chinese Academy of Sciences (GSA: CRA008871) and are publicly accessible at https://ngdc.cncb.ac.cn/gsa. Qualified libraries were sequenced using an Illumina NovaSeq 6000 system in a paired-end format. Reads were mapped to the reference genome (Rnor_6.0.104, ENSEMBL) using Hisat2 and feature counts were used to count the reads. Fragments per kilobase million were calculated after normalization to the trimmed mean of M values. The edgeR package was used to analyze significantly differentially expressed genes. Gene set enrichment analysis (GSEA) was performed using the pre-ranked method in GSEA Java implementation (26). The WIKI pathway database (MsigDB, http://software.broadinstitute.org/gsea/) was used for gene annotation, and a p-value <0.05 was considered statistically significant.

26. Subramanian A, Tamayo P, Mootha VK, Mukherjee S, Ebert BL, Gillette MA, et al. Gene Set Enrichment Analysis: A Knowledge-Based Approach for Interpreting Genome-Wide Expression Profiles. Proceedings of the National Academy of Sciences of the United States of America (2005) 102(43):15545-15550. doi: 10.1073/pnas.0506580102.
